# Radiologic–pathologic correlation in meningeal melanocytosis: A case report

**DOI:** 10.1097/MD.0000000000048881

**Published:** 2026-05-15

**Authors:** Dong Bai, Fang Li, Zhiqun Wang

**Affiliations:** a Department of Radiology, Aerospace Center Hospital, Beijing, China.

**Keywords:** case report, leptomeningeal enhancement, melanocytic tumors, meningeal melanocytosis, radiologic–pathologic correlation

## Abstract

**Rationale:**

Meningeal melanocytosis is an exceedingly rare diffuse leptomeningeal melanocytic disorder that frequently mimics meningeal carcinomatosis or chronic infectious meningitis on conventional magnetic resonance imaging (MRI), posing substantial diagnostic challenges. This report aims to enhance clinical awareness of this condition and underscore the indispensable role of radiologic–pathologic correlation in achieving a definitive diagnosis.

**Patient concerns:**

A 19-year-old male presented with a 3-month history of progressive headache, nausea, vomiting, and one episode of generalized tonic-clonic seizure. Neuroimaging demonstrated diffuse linear leptomeningeal enhancement without restricted diffusion or conspicuous T1 shortening. Cerebrospinal fluid (CSF) analysis, bacterial and fungal cultures, next-generation sequencing (NGS), and positron emission tomography–computed tomography (PET-CT) were all non-diagnostic, and empirical anti-infective therapy failed to halt disease progression.

**Diagnoses:**

Following comprehensive exclusion of infectious, inflammatory, autoimmune, and neoplastic etiologies, stereotactic leptomeningeal biopsy was performed. Histopathology revealed diffuse proliferation of cytologically bland melanocytes within the leptomeninges; immunohistochemistry confirmed S-100, HMB45, and Melan-A positivity with a Ki-67 proliferative index of approximately 1% and wild-type BRAF V600E status, establishing a definitive diagnosis of meningeal melanocytosis.

**Interventions:**

The patient was treated with intravenous dexamethasone (10 mg/day) to manage cerebral edema and levetiracetam (500 mg twice daily) for seizure prophylaxis. Referral to a specialized neuro-oncology center was arranged for evaluation of definitive therapy, including potential whole-brain radiotherapy or intrathecal chemotherapy.

**Outcomes:**

Symptoms improved markedly following biopsy and initiation of corticosteroid therapy; headaches resolved, vomiting ceased, and no further seizures occurred during hospitalization. The patient was discharged neurologically intact on day 15 with a healed surgical wound, though long-term follow-up imaging was unavailable due to loss to specialized follow-up.

**Lessons:**

Meningeal melanocytosis may present without classic melanin-related T1 hyperintensity, rendering it indistinguishable from carcinomatosis on standard MRI sequences. When noninvasive investigations including CSF studies, NGS, and PET-CT are inconclusive, timely tissue biopsy is warranted. Incorporating susceptibility-weighted imaging and molecular testing for GNAQ/GNA11 mutations into the diagnostic workup may improve early and accurate identification of this rare condition.

## 1. Introduction

Meningeal melanocytosis belongs to the spectrum of primary meningeal melanocytic neoplasms – a group of rare tumors arising from melanocytes that normally reside in the leptomeninges.^[1]^ The 2021 WHO Classification of Central Nervous System Tumors divides these neoplasms into diffuse forms (melanocytosis and melanomatosis) and localized forms (melanocytoma and melanoma).^[2]^ Melanocytosis, the benign diffuse variant, is characterized by widespread proliferation of cytologically bland melanocytes within the pia and arachnoid, typically without invasion of the brain parenchyma, though extension along Virchow–Robin spaces has been documented.^[1,3]^

Clinically, patients with meningeal melanocytosis tend to present in childhood or young adulthood with nonspecific complaints – headache, nausea, seizures, cranial nerve deficits – that overlap considerably with infectious meningitis, neurosarcoidosis, and leptomeningeal carcinomatosis.^[4,5]^ On magnetic resonance imaging (MRI), diffuse leptomeningeal enhancement is the principal finding; however, when melanin-related T1 shortening is absent, this pattern becomes virtually indistinguishable from carcinomatous or inflammatory meningitis.^[6]^ Although susceptibility-weighted imaging (SWI) and quantitative T1 mapping have been proposed to improve diagnostic specificity, experience with these techniques in melanocytic lesions remains limited.^[7]^

Because of this substantial imaging overlap, histopathological confirmation through meningeal or brain biopsy often remains the only definitive diagnostic route. Here, we report a 19-year-old male who presented with progressive diffuse leptomeningeal enhancement initially raising concern for meningeal carcinomatosis; stereotactic biopsy confirmed meningeal melanocytosis. We describe the radiologic–pathologic correlation, address the differential diagnosis, and review the molecular and prognostic features of diffuse meningeal melanocytic neoplasms.

## 2. Case report

### 2.1. Clinical presentation and initial workup

A 19-year-old male college student was admitted to our hospital on February 14, 2023, with a 3-month history of intermittent headache and vomiting. The headaches had begun insidiously without a clear trigger and were accompanied by nausea and non-bilious vomiting. During the illness, he experienced one episode of generalized tonic-clonic seizure with transient loss of consciousness and limb convulsions, which resolved spontaneously within approximately 2 minutes. He had no prior neurological history and no family history of melanoma, neurocutaneous syndromes, or congenital melanocytic nevi.

At a local hospital, he received dehydration therapy and corticosteroids, which partially alleviated his symptoms. MRI at that facility showed leptomeningeal enhancement, and infectious meningitis was initially considered. Magnetic resonance angiography was unremarkable. Lumbar puncture revealed an opening pressure of 280 mm H_2_O; cerebrospinal fluid (CSF) routine analysis, biochemistry, cytology, and bacterial and fungal cultures were all within normal limits. Despite empirical anti-infective therapy (details of the regimen were unavailable), leptomeningeal enhancement progressed on follow-up imaging. A subsequent positron emission tomography–computed tomography (PET-CT) at another institution demonstrated diffuse fluorodeoxyglucose uptake along the leptomeninges without focal hypermetabolic parenchymal lesions; the interpreting radiologist raised concern for meningeal carcinomatosis and recommended brain biopsy.

### 2.2. Admission findings

On admission to our center, the patient was alert but fatigued. Neurological examination was unremarkable: pupillary reflexes were normal and symmetric, motor strength was 5/5 in all extremities, sensory examination was intact, and no pathological reflexes were elicited. Notably, there were no cutaneous melanocytic lesions, congenital nevi, or other findings suggestive of neurocutaneous melanosis (e.g., giant congenital melanocytic nevi).

**Figure 1.**
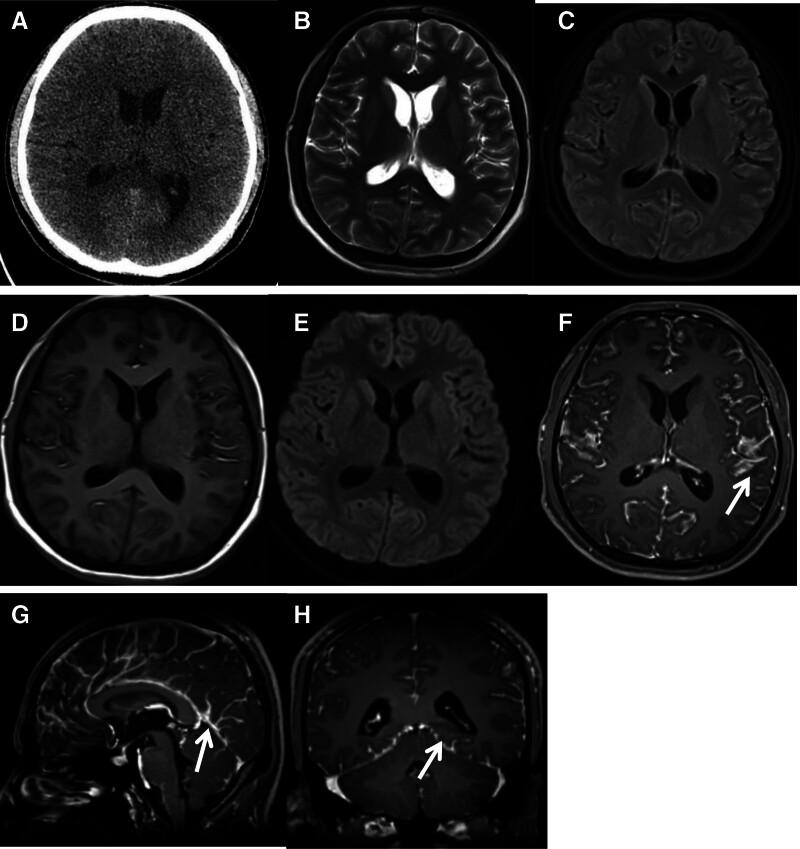
Radiologic findings. (A) Plain cranial CT: no abnormal density. (B–C) T2WI and FLAIR: subtle cortical hyperintensity. (D) T1WI: linear structures within the cerebral sulci of both hemispheres; definitive T1 shortening attributable to melanin was not conspicuous. (E) DWI: no restricted diffusion. (F–H) Post-contrast T1WI: diffuse linear and strip-like leptomeningeal enhancement (arrow). FLAIR = fluid-attenuated inversion recovery.

Cranial CT (Fig. 1A) showed no definite abnormalities. Brain MRI (Fig. 1B–H) demonstrated subtle cortical hyperintensity on fluid-attenuated inversion recovery and T2-weighted sequences without restricted diffusion on diffusion-weighted imaging; post-contrast images revealed diffuse linear leptomeningeal enhancement involving both cerebral hemispheres. On unenhanced T1-weighted images (Fig. 1D), several linear structures were noted within the cerebral sulci; however, definitive T1 hyperintensity, commonly associated with melanin-containing lesions, was not prominent. SWI was not acquired during this examination. Next-generation sequencing of CSF detected no tumor-derived mutations. Considering all findings, a diffuse leptomeningeal process was favored; meningeal carcinomatosis remained in the differential.

### 2.3. Biopsy and histopathological findings

**Figure 2.**
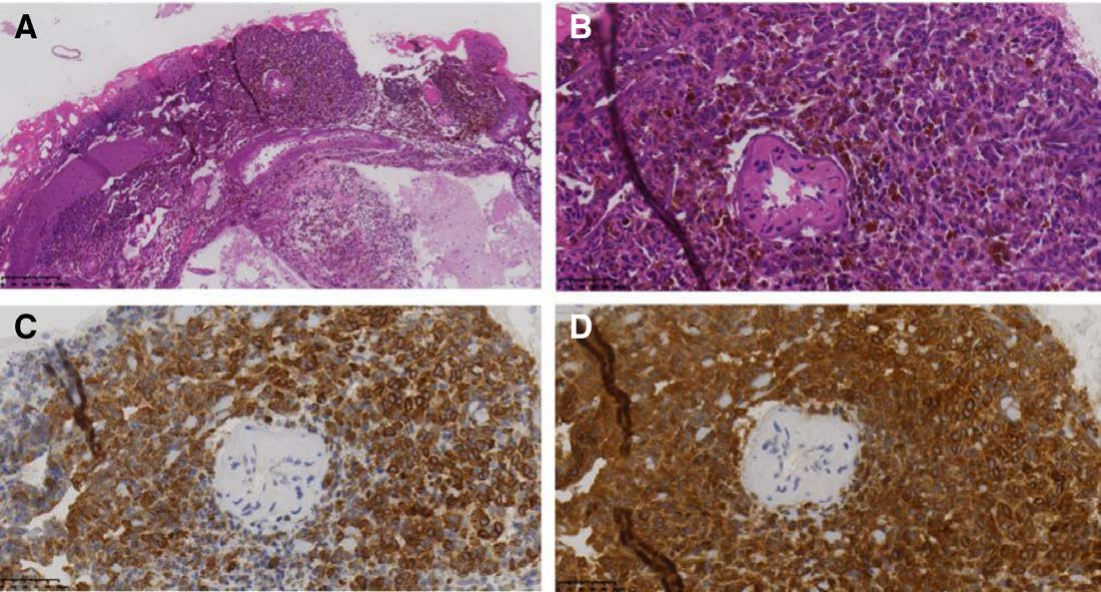
Histopathologic findings. (A) H&E staining (×100): diffuse melanocyte proliferation with pigment deposition in the leptomeninges. (B) High-power view (×400): polygonal cells with uniform nuclei and no mitotic activity. (C–D) Immunohistochemistry: HMB45 and Melan-A positivity confirming melanocytic differentiation. H&E = hematoxylin and eosin.

On February 16, 2023, the patient underwent stereotactic biopsy of the leptomeningeal lesion under general anesthesia. Intraoperatively, the arachnoid membrane beneath the dura appeared diffusely dark-brown to black, and representative tissue was obtained from the abnormal meninges. Histopathological examination (Fig. 2) showed diffuse proliferation of polygonal melanocytes within the leptomeninges, with abundant intracytoplasmic melanin pigment. Cells exhibited small, round, uniform nuclei without prominent nucleoli, and no mitotic figures were identified. Perivascular spaces within the underlying brain parenchyma also contained melanocytic proliferation, consistent with extension along Virchow–Robin spaces rather than true parenchymal invasion. No necrosis or desmoplastic reaction was present.

Immunohistochemistry demonstrated strong and diffuse positivity for S-100, HMB45, and Melan-A, confirming melanocytic lineage. The Ki-67 proliferative index was approximately 1%, indicating low proliferative activity. INI1 nuclear expression was retained (ruling out atypical teratoid/rhabdoid tumor), and epithelial membrane antigen and desmin were negative. SOX10 staining was performed but was noncontributory because heavy melanin pigmentation obscured the signal. BRAF V600E mutation testing by immunohistochemistry was negative (wild type). Molecular testing for GNAQ, GNA11, and NRAS mutations was not available at our institution at the time of diagnosis; these mutations represent common oncogenic drivers in primary leptomeningeal melanocytic neoplasms and would have been informative both for confirming the diagnosis and for guiding potential targeted therapy.^[8,9]^

Combining the histopathological features – diffuse leptomeningeal melanocytic proliferation, bland cytology, absent mitoses, low Ki-67, and wild-type BRAF V600E – with the imaging pattern of diffuse leptomeningeal enhancement, the diagnosis was consistent with meningeal melanocytosis (World Health Organization grade not formally assigned to this entity in the 2021 classification).

### 2.4. Treatment and follow-up

Postoperatively, the patient received intravenous dexamethasone (10 mg/day) for cerebral edema and was started on levetiracetam (500 mg twice daily) for seizure prophylaxis. Symptoms improved markedly: headaches diminished, vomiting ceased, and no further seizures occurred during hospitalization.

The patient was discharged on March 3, 2023, in good general condition. At discharge, he was alert, oriented, and neurologically intact; the surgical wound had healed without complication. A follow-up MRI was scheduled for 3 months post-discharge, and referral to a specialized neuro-oncology center was arranged for discussion of definitive therapy, potentially including whole-brain radiotherapy or intrathecal therapy.

By telephone follow-up, however, we learned that the patient did not attend the scheduled neuro-oncology consultation. Instead, he returned to his local hospital and continued symptomatic treatment with tapering corticosteroids and levetiracetam. No follow-up MRI was obtained. This lack of longitudinal imaging and clinical outcome data constitutes a major limitation of this report. Because meningeal melanocytosis can follow a variable clinical course – from indolent stability to progressive neurological deterioration and, in some cases, malignant transformation – ongoing surveillance is critical, and we are unable to draw conclusions about this patient’s long-term outcome^[1,3,10]^ (Table 1).

**Table 1:** Patient timeline.

Date	Event	Key findings/ Actions
November 2022	Symptom onset: intermittent headache, vomiting	One episode of generalized tonic-clonic seizure
November–December 2022	Evaluation at local hospital	MRI: leptomeningeal enhancement; LP: opening pressure 280 mm H_2_O, CSF unremarkable; empirical antibiotics given
January 2023	PET-CT at referral hospital	Diffuse FDG uptake along leptomeninges; concern for meningeal carcinomatosis; biopsy recommended
February 14, 2023	Admission to our center	CT: normal; MRI: diffuse leptomeningeal enhancement, no T1 shortening, no diffusion restriction; CSF NGS: negative
February 16, 2023	Stereotactic biopsy	Dark arachnoid; pathology: melanocytic proliferation, Ki-67 ~1%, HMB45+/Melan-A+, BRAF V600E wild type
February 17–March 3, 2023	Postoperative management	Dexamethasone 10 mg/day IV; levetiracetam 500 mg BID; symptoms improved
March 3, 2023	Discharge	Good condition; follow-up MRI and neuro-oncology referral scheduled
Post-discharge	Telephone follow-up	Patient returned to local hospital; no follow-up MRI or neuro-oncology visit; continued symptomatic treatment

BID = twice daily, CSF = cerebrospinal fluid, CT = computed tomography, FDG = fluorodeoxyglucose, HMB45 = human melanoma black 45, IV = intravenous, Ki-67 = proliferation index, LP = lumbar puncture, MRI = magnetic resonance imaging, NGS = next-generation sequencing, PET-CT = positron emission tomography–computed tomography.

## 3. Discussion

This case highlights the diagnostic challenge posed by meningeal melanocytosis when it manifests as diffuse leptomeningeal enhancement on MRI without the expected T1 hyperintensity. In our patient, the clinical and imaging picture closely resembled meningeal carcinomatosis, and stereotactic biopsy was the only means of resolving the diagnostic uncertainty.

The differential diagnosis of diffuse leptomeningeal enhancement is broad, encompassing infectious meningitis (tuberculous, fungal, viral), neurosarcoidosis, autoimmune meningitis (e.g., IgG4-related disease, rheumatoid meningitis), leptomeningeal carcinomatosis, and primary meningeal melanocytic neoplasms.^[5,6,11]^ In our patient, infectious etiologies were deemed unlikely because CSF cultures, cytology, and biochemistry were repeatedly normal and symptoms progressed despite empirical antimicrobial therapy. Autoimmune or granulomatous causes such as neurosarcoidosis were not formally investigated with serum angiotensin-converting enzyme or chest imaging, which we acknowledge as a limitation; however, PET-CT did not reveal hilar lymphadenopathy or other systemic findings suggestive of sarcoidosis.

The PET-CT finding of diffuse leptomeningeal fluorodeoxyglucose uptake without focal parenchymal hypermetabolism is compatible with both carcinomatous and melanocytic leptomeningeal involvement and lacks sufficient specificity to discriminate between these entities.^[12]^ This appropriately led to the decision to pursue tissue diagnosis.

Among melanocytic lesions, melanin is paramagnetic and classically produces T1 hyperintensity on unenhanced MRI – a finding considered characteristic but by no means invariable.^[6,13]^ Several factors may explain the absence of conspicuous T1 shortening in our case. The melanocytes were diffusely distributed in a thin layer across the leptomeninges, and the melanin concentration per voxel may have been below the detection threshold at 3T. Additionally, the degree of melanin content varies substantially among meningeal melanocytic neoplasms; amelanotic or pauci-melanotic variants have been described in localized melanocytomas and melanomas, and this variability likely extends to diffuse melanocytosis.^[14]^ Furthermore, SWI – which is sensitive to the paramagnetic effects of melanin and may reveal melanin-laden deposits even when T1 shortening is absent – was not performed in this case and might have provided supplementary diagnostic information.^[7]^ Quantitative T1 mapping, another potentially useful technique, was likewise unavailable. These findings emphasize the importance of a multisequence MRI protocol, including SWI, when evaluating unexplained leptomeningeal enhancement in young patients.

In comparison with previously reported cases, our patient’s age (19 years) falls within the typical range for meningeal melanocytosis, which peaks in children and young adults.^[1,3]^ Unlike cases associated with neurocutaneous melanosis – in which giant congenital melanocytic nevi provide a clinical clue – our patient had no cutaneous findings, which made pre-biopsy suspicion for a melanocytic process especially low.^[15]^

The histopathological distinction between meningeal melanocytosis and its malignant counterpart, meningeal melanomatosis, rests on cytological features, mitotic activity, and growth pattern.^[2]^ In our case, bland cytology, absent mitoses, low Ki-67 (≈1%), and lack of necrosis or parenchymal invasion all supported a diagnosis of melanocytosis rather than melanomatosis. It should be acknowledged, however, that sampling bias inherent in stereotactic biopsy may underestimate heterogeneous areas of higher-grade transformation, and distinguishing between these entities on limited tissue can be challenging.^[16]^

At the molecular level, primary meningeal melanocytic neoplasms frequently harbor activating mutations in GNAQ or GNA11 (reported in up to 50% to 80% of circumscribed tumors), which encode G-protein alpha subunits in the MAP kinase signaling pathway; NRAS mutations are more common in diffuse forms, especially those associated with neurocutaneous melanocytosis in children.^[8,9]^ BRAF V600E mutations, prevalent in cutaneous melanoma, are generally absent in primary central nervous system melanocytic tumors – a finding corroborated in our patient.^[8]^ The molecular profile serves dual purposes: it aids in distinguishing primary meningeal neoplasms from leptomeningeal metastasis of cutaneous melanoma, and it may open therapeutic avenues, as MEK inhibitors have shown activity in GNAQ/GNA11-mutant melanocytic tumors in small series.^[17]^ Molecular testing for GNAQ, GNA11, and NRAS was unavailable at our institution at the time, which we recognize as a shortcoming of our workup.

No consensus guidelines currently exist for the treatment of meningeal melanocytosis. In asymptomatic or mildly symptomatic patients, observation with serial MRI has been adopted.^[1,3,10]^ For patients with progressive symptoms or extensive leptomeningeal disease, reported options include whole-brain radiotherapy, focal radiation, intrathecal chemotherapy, systemic targeted therapy (MEK inhibitors for GNAQ/GNA11-mutant cases), and more recently, immune checkpoint inhibitors.^[10,17,18]^ In our patient, initial symptomatic management with corticosteroids and antiepileptics was a reasonable approach given his rapid clinical improvement after biopsy; however, definitive therapy was deferred pending evaluation at a neuro-oncology center, which the patient unfortunately did not attend.

The prognosis of meningeal melanocytosis varies widely. Some patients remain stable for years, while others develop progressive hydrocephalus, malignant transformation to melanomatosis, or widespread central nervous system dissemination.^[1,3,10]^ In the recent EURACAN review by Pellerino et al,^[1]^ treatment data for primary diffuse leptomeningeal melanocytic neoplasms remain sparse, and the optimal therapeutic approach is still unclear. The absence of follow-up data in our case prevents any assessment of disease trajectory.

The value of this report lies in the detailed radiologic–pathologic correlation demonstrating how meningeal melanocytosis can closely mimic carcinomatosis when classic melanin-related MRI features are absent. At the same time, several limitations must be noted. SWI and quantitative T1 mapping were not acquired. Molecular testing beyond BRAF V600E was not performed. The patient was lost to specialized follow-up, which prevents us from assessing disease evolution or treatment response. Future cases should incorporate advanced MRI sequences and comprehensive molecular profiling to refine the diagnostic algorithm.

## 4. Conclusions

Meningeal melanocytosis should be considered in the differential diagnosis of diffuse leptomeningeal enhancement, even in the absence of T1 hyperintensity, particularly in young patients without an identifiable primary malignancy. When noninvasive investigations – including CSF analysis, next-generation sequencing, and PET-CT – do not yield a definitive diagnosis, timely biopsy is justified to prevent misdiagnosis and guide further management. Incorporating SWI and molecular testing for GNAQ/GNA11 mutations may help refine the diagnostic workup.

## Acknowledgements

We thank the Department of Neurosurgery for technical support during diagnosis and treatment, and the patient for active cooperation in this study.

## Author contributions

**Conceptualization:** Dong Bai, Fang Li, Zhiqun Wang.

**Resources:** Fang Li.

**Writing – original draft:** Dong Bai.

**Writing – review & editing:** Zhiqun Wang.
